# Association of National Area Deprivation Index with dietary patterns and pain-related inflammatory biomarkers in community-dwelling adults with chronic low back pain

**DOI:** 10.3389/fmscd.2025.1601314

**Published:** 2025-10-21

**Authors:** Larissa J. Strath, Jonas G. Dembowski, Javier A. Tamargo, Andrew M. Sims, Demario S. Overstreet, Terence M. Penn, Rahm J. Bakshi, Eeshaan Bajaj, Tammie L. Quinn, D. Leann Long, Robert E. Sorge, Burel R. Goodin

**Affiliations:** 1 Department of Health Outcomes and Biomedical Informatics, University of Florida, Gainesville, FL, United States; 2 Pain Research and Intervention Center of Excellence, University of Florida, Gainesville, FL, United States; 3 Institute on Aging, University of Florida, Gainesville, FL, United States; 4 Department of Psychology, The University of Alabama at Birmingham, Birmingham, AL, United States; 5 Department of Biostatistics, The University of Alabama at Birmingham, Birmingham, AL, United States; 6 Department of Surgery, The University of Alabama at Birmingham, Birmingham, AL, United States; 7 Harvard Medical School and Massachusetts General Hospital, Boston, MA, United States; 8 Department of Anesthesiology, Washington University at St. Louis, ST. Louis, MO, United States

**Keywords:** pain, low back pain, diet, socioeconomic disparity, Area Deprivation Index (ADI)

## Abstract

**Introduction:**

Chronic low back pain (cLBP) is a common health condition associated with substantial personal and economic costs. Recent literature suggests that socioeconomic status (SES) and diet quality may influence its impact.

**Methods:**

The purpose of this study was to examine whether SES, measured via the National Area Deprivation Index (NADI), and diet quality, assessed by the dietary inflammatory index (DII), were associated with proinflammatory cytokine levels and movement-evoked pain outcomes in individuals with cLBP. We hypothesized that individuals with cLBP with lower NADI and DII scores would exhibit significantly greater pain and higher levels of inflammatory biomarkers. Participants with cLBP (*n* = 78) completed questionnaires assessing pain and demographic factors, along with a 24 h food recall. Inflammatory biomarkers were measured from peripheral blood samples collected prior to the completion of the questionnaires.

**Results:**

Analyses revealed that NADI and DII were associated with a similar profile of inflammatory biomarkers and pain outcomes and that DII varied as a function of NADI.

**Conclusions:**

These findings offer important information for future targeting goals for treating vulnerable populations with cLBP. Future studies are warranted to determine whether the relationships among SES, diet quality, and inflammation extend to other chronic pain conditions.

## Introduction

Chronic pain is a significant health condition that imposes substantial physical, psychosocial, and financial burdens on individuals and communities (1). The International Association for the Study of Pain describes pain as “an unpleasant sensory and emotional experience associated with, or resembling that associated with, actual or potential tissue damage” (2). Chronic pain can lead to anxiety, depression, and disruptions in daily activities, including the ability to care for oneself or one’s family, all of which require time and financial resources (3). Among the various types of chronic pain, chronic low back pain (cLBP) is one of the most common. In adults, the prevalence of cLBP has increased by more than 100% in the last 10 years (4). Like other forms of chronic pain, cLBP is considered to have recurring or persistent symptoms lasting longer than 3 months (3). This painful disorder can be classified into two subgroups: specific cLBP and idiopathic cLBP. In specific cLBP, there is a tangible underlying etiology, making treatment modalities easier to determine and implement (5). In contrast, the vast majority of cLBP cases are idiopathic, in which the exact pathological source of pain remains unknown (5). As the majority of cLBP cases fall under this category, most monotherapy treatments are ineffective and poor at best due to the lack of understanding of its root cause (6).

Several risk factors are associated with cLBP, including demographic, psychological, and environmental factors (7). Recent literature has identified lower socioeconomic status (SES) as a risk factor not only for the development of chronic pain but also for greater pain severity (8). Low SES is characterized by a lack of access to social, educational, financial, and healthcare resources, while both medium and high SES are associated with progressively greater access (9). Used together as an overall SES variable, as well as independently, lower levels of education, income inequality, and higher levels of deprivation are all associated with increased cLBP prevalence (10). It is well-established that individuals with lower SES are likely to hold multiple jobs, especially those that require manual labor, which increases their risk of developing cLBP compared to non-manual laborers due to physical stress (7, 11). In addition, it has been reported that limited access to healthcare, lack of access to gyms/physical activity, and food insecurity may all play a role in influencing the development of painful disorders such as cLBP.

An inflammatory response is characteristic of individuals with cLBP (12), and this can be influenced by behavioral factors such as dietary patterns. Current literature has shown that healthier eating patterns are related to lower levels of proinflammatory biomarkers and higher levels of anti-inflammatory biomarkers (13). Specifically, foods such as fish, whole grains, fruits, and vegetables are recognized for their anti-inflammatory properties (13). Additional evidence suggests that an unbalanced diet (characterized by excess consumption of refined sugars, saturated fatty acids, and free radicals, along with deficiencies in vitamins, minerals, and antioxidants) can influence pain conditions, possibly through inflammation (14). Unfortunately, healthier food options are less accessible in low-SES areas (15), leading individuals in these areas to rely more heavily on less healthy, calorie-dense, processed foods. In 2019, a systematic review of the literature on obesity (a proinflammatory condition) and national SES found that individuals with low SES had a 45% higher odds of obesity and a 31% higher odds of being overweight (16). Similarly, diets high in processed and fried foods have been associated with greater reports of pain than plant-based diets (17).

As stated, treatment options for idiopathic cLBP are mediocre because they (1) often fail to reduce pain severity and (2) come with a negative side-effect profile that creates additional health burdens. Therefore, there is an urgent need to develop effective treatment modalities with a better side-effect profile. Because diet quality, like many pharmaceuticals, can perpetuate and decrease inflammation and has a side-effect-free profile, it may be efficacious to harness it as a therapeutic option for cLBP treatment. However, factors influencing diet quality must also be accounted for when developing such treatments. The purpose of this study was to examine levels of inflammatory biomarkers in participants with idiopathic cLBP in relation to their SES and diet quality [as measured via the dietary inflammatory index (DII)] to determine a relationship among these three variables to lay the groundwork for potential future studies and interventions.

## Methods and materials

### Participants

Community-dwelling adults (*n* = 78) with idiopathic cLBP were recruited from the Birmingham, Alabama, geographical area. Participants were evaluated by a telephone screening to provide general information about their chronic pain and medical history to determine eligibility. Participants were asked to self-identify their race, ethnicity, sex, and gender. Inclusion criteria required persistent lower back pain lasting 6 months or longer. Eligible participants were aged 19–85 years. Excluding criteria consisted of known (i.e., specific) cLBP etiology, back surgery within the last year, accident or trauma in the previous year, uncontrolled blood pressure, heart disease, systemic lupus, cancer, diabetes, ankylosing spondylitis, multiple sclerosis, stroke, epilepsy, fibromyalgia, Raynaud’s disease, major depression/bipolar disorders, and HIV. Screening and eligibility procedures were conducted in accordance with approval from the University of Alabama at Birmingham (UAB) Institutional Review Board (#170119003). All participants provided written informed consent that has been approved by the UAB IRB and conducted in accordance with the Declaration of Helsinki.

### Twenty-four-hour food recall

A 24-h food recall questionnaire was delivered to acknowledge the typical food consumption of participants over a single day. Trained research assistants conducted the recall and probed participants for additional ingredients that may have been missed. The recall included all meals from the first meal to the last meal, along with any snacks and beverages consumed in the 24 h prior to experimental testing.

### Nutritional analysis

The Nutrition Data System for Research 2020 (NDSR; University of Minnesota, Minneapolis, MN, USA) was used to analyze all nutritional data. The 24-h food recall data were entered into NDSR, and a complete list of macro- and micronutrients was generated.

The DII was then calculated as per the protocol in SPSS version 29 (57). For this study, the DII was calculated using the following components: alcohol; vitamins A, B, B12, C, D, and E; total carbohydrates; total fat; cholesterol; fiber; folic acid; iron; magnesium; beta-carotene; energy (kcal); monounsaturated and polyunsaturated fatty acids; N-3 and N-6 fatty acids; protein; riboflavin; saturated fatty acids (SFA); selenium; thiamin; zinc; trans fats; caffeine; and isoflavones. The DII score ranges from positive to negative values, with positive values indicating higher levels of inflammatory nutrients and negative values indicating a greater presence of anti-inflammatory nutrients.

### National Area Deprivation Index

To quantify SES, the National Area of Deprivation Index (NADI) was used to provide data about disadvantages based on one’s residence. Participants’ self-reported addresses from the demographic questionnaire were used to determine their nine-digit zip codes via UnitedStateszipcode.org. These zip codes were then processed in MATLAB (version 9.9) by loading the code zippy.m to generate scores ranging from 0 to 100, with higher scores indicating greater levels of neighborhood disadvantage. The scores are calculated based on education, employment, income, and housing quality (18).

### Blood-based biomarkers

A single blood draw was performed at session commencement by a trained phlebotomist. Blood was collected into 4 mL purple-top EDTA tubes and centrifuged at 1,500–1,800*g* (rcf) for 10 min; plasma was then removed and stored at −80°C for later analysis. The collected blood was used to assess numerous biomarkers, including interferon gamma (IFN-γ); interleukins (ILs): IL-10, IL-12p70, IL-13, IL-1-β, IL-2, IL-4, IL-6, IL-8, IL-15, IL-16, IL-17A, IL-1-α, IL-5, IL-23p40, and IL-7; tumor necrosis factors (TNFs): TNF-α and TNF-β; eotaxin and eotaxin-3; monocyte chemoattractant proteins (MCPs): MCP-1 and MCP-4; macrophage-derived chemokine (MDC); macrophage inflammatory proteins (MIPs): MIP-1α and MIP-1β; thymus and activation-regulated chemokine (TARC); granulocyte-macrophage colony-stimulating factor (GM-CSF); interferon gamma-inducible protein 10 (IP-10); and vascular endothelial growth factor 5 (VEGF-5).

### Functional performance and short physical performance battery

#### Bed task

Participants were instructed to stand next to the bed and simulate their normal method of getting into bed. Once lying flat on their back, they were instructed to get out of bed as they normally would and stand beside it. This exercise was repeated twice, and after the second trial, participants reported their pain and difficulty on a scale from 0 to 100.

#### Box-lift tests

Female participants were instructed to lift a 9-pound box and place it on a 30-in table, while male participants were asked to lift a 14-pound box and place it on a 30-in table. After placing the box on the table, all participants were instructed to return it to the floor in front of them. This sequence was repeated four consecutive times. After the final lift, participants rated their pain and difficulty again on a 0–100 scale. At any point during the test, the test was stopped immediately if a participant was unable to lift the box.

#### SPPB: balance tests

To begin the short physical performance battery (SPPB), participants were asked to complete a balance test consisting of three parts, each with a different stance. The first was the side-by-side, followed by semi-tandem, ending with full-tandem. Participants scored 0–1 on the side-by-side and semi-tandem tests. A score of 1 indicates that the participant held the position for 10 s, while 0 indicates that the participant did not make it to 10 s or could not complete the task. The full-tandem test was scored on a 0–2 scale, with 2 indicating holding the position for 10 s, 1 indicating maintaining the position for 3–9.99 s, and 0 indicating holding it for less than 3 s or not completing the test. In the end, scores from all stances were summed, and participants were asked to rate their pain and difficulty with the balance tests.

#### SPPB: gait speed tests

Participants were instructed to walk a 4-m course twice at their normal walking speed. The timing was recorded, and walking aids were permitted if necessary. The faster of the two times was used for scoring. Scores ranged from 0 to 4, with 0 indicating that the test was not done, 1 indicating that the participant took longer than 8.70 s, 2 indicating 6.21–8.7 s, 3 corresponding to 4.82–6.21 s, and 4 indicating less than 4.82 s. In the end, participants were asked to rate their pain and difficulty on a 0–100 scale.

#### SPPB: chair stands

Participants were instructed to rise from a chair without using their hands. If successful, they were then asked to complete five consecutive chair stands, all without using their arms. They were told to complete the task as quickly as possible, and the time was recorded. Scores ranged from 0 to 4, with 0 indicating that the participant was unable to complete the task at all or required more than 60 s, 1 indicating that the chair stands were finished in 16.70–60 s, 2 indicating that the chair stands were finished in 13.70–16.70 s, 3 indicating that the chair stands were finished in 11.20–13.70 s, and finally 4 indicating that the chair stands were finished in less than 11.20 s. After the chair stands were completed, participants were asked to rate their pain and difficulty on a 0–100 scale.

#### Timed up-and-go

The final movement task was the timed up-and-go (TUG). Participants were asked to start the test sitting in a chair. When ready, they were instructed to rise from the chair, walk a 3-m course at a normal walking pace, turn around at the end of the course, return to their chair, and sit down. Time and self-reported pain and difficulty were recorded.

### Statistical analyses

All statistical analyses were performed using IBM SPSS Statistics, version 29. Prior to analyses, the data were cleaned and tested for normality using Levine’s test and skewness and kurtosis assessments. To examine potential demographic differences, chi-square tests and analyses of variance (ANOVAs) were conducted as appropriate. Participants were categorized into three groups based on NADI scores: high (66–100), medium (33–66), and low (0–33). Partial correlation analyses were also performed to determine associations among DII, blood biomarkers, and pain outcomes. Correlations were also conducted between NADI as a continuous variable and blood biomarkers. In addition, we performed analyses of covariance (ANCOVAs) with the NADI groups as the fixed factor and DII, pain, and physical function outcomes as the dependent variables. Age, sex, race, income, education, caloric intake, and BMI were controlled for due to their potential confounding effects on DII and/or pain outcomes. Finally, a *post-hoc* power analysis was conducted for sensitivity. The significance level was set at *p* ≤ 0.05 in all cases.

## Results

### Participant characteristics

Of the 279 individuals with cLBP who participated in the study, only 78 had valid DII scores, NADI scores, biomarker data, and covariate data available for analyses. Of the entire sample, most participants were women (57.7%) and non-Hispanic Black (64.1%), with a mean age of 44.71 (±12.76) years. The mean NADI score was 69.08 (±26.803), and most participants had partial college education or less (68%). The average BMI was 30.87 (±8.060), and the average DII score was 2.10 (±1.756). A complete breakdown of participant demographic characteristics stratified by the NADI group is presented in Table 1. NADI, DII, the biomarkers IL-1B, MDC, MIP-1α, MIP-1β, IL-12, IL-15, and TNF-β showed normal and homogenous distribution. All other variables were log_10_-transformed for parametric analyses.

### Correlation analyses results

Using Pearson correlation analyses, the relationship between DII and biomarkers was found. DII was positively correlated with TNF-α (*r*^2^ = 0.206, *p* = 0.050) and showed a positive trend with MDC (*r*^2^ = 0.368, *p* = 0.071). Similarly, the relationship between DII and pain outcomes was also found. DII was positively correlated with balance pain (*r*^2^ = 0.206, *p* = 0.049) and chair-stand pain (*r*^2^ = 0.192, *p* = 0.050), with a positive trend emerging for bed-task pain (*r*^2^ = 0.206, *p* = 0.073). NADI showed significant correlation with several biomarkers, such as IL-12p70 (*r*^2^ = −0.514, *p* = 0.01), IL-12 (*r*^2^ = −0.0449, *p* = 0.028), IL-2 (*r*^2^ = −0.400, *p* = 0.050), MCP4 (*r*^2^ = −0.426, *p* = 0.038), and IL-7 (*r*^2^ = −0.640, *p* = 0.001).

### NADI group analyses

Complete descriptive statistics of the variables that statistically differed by the NADI group is provided in Table 2.

#### Biomarkers and DII

**FIGURE 1:**
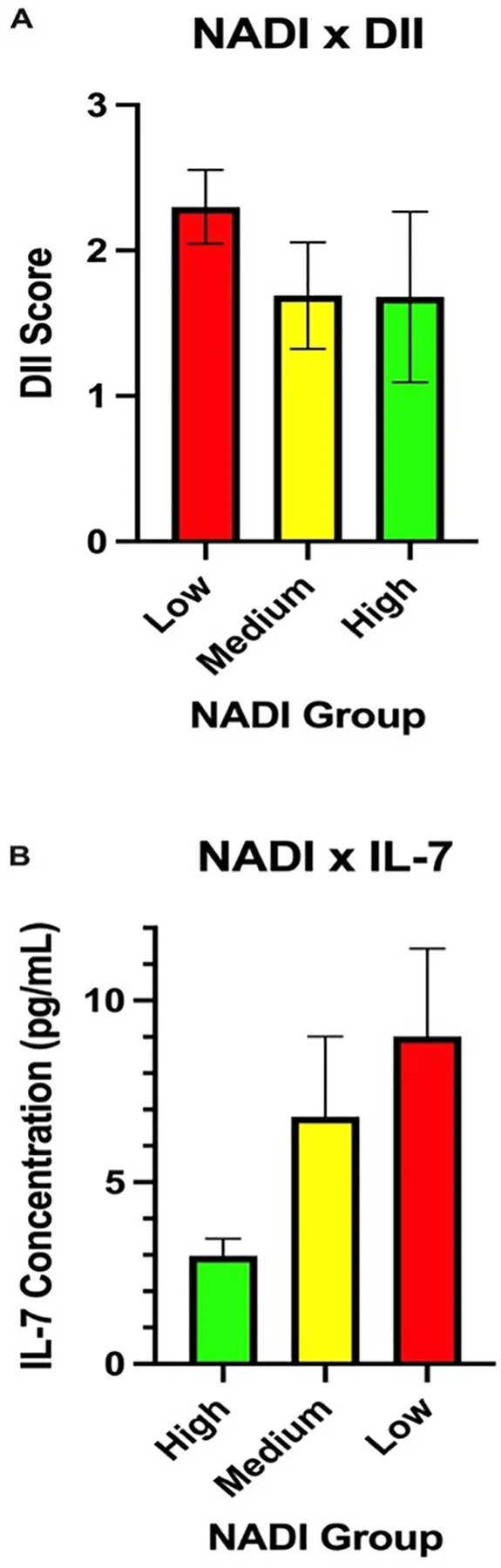
**(A)** DII scores among the three NADI groups; **(B)** IL-7 scores among the three NADI groups.

A significant difference in DII levels [*F*(2,76) = 179.48, *p* = 0.006] was observed between the NADI groups (high, medium, low), with the mean averages for the three groups shown in Figure 1A. Similarly, several biomarkers, such as TNF-β [*F*(2,24) = 26.84, *p* = 0.036], IP-10 [*F*(2,74) = 25.6, *p* = 0.038)], and IL-7 [*F*(2,24) = 73.76, *p* = 0.013], showed significant differences across the three NADI groups. The average values for biomarker IL-7 across the three NADI groups are shown in Figure 1B.

#### Pain outcomes

**FIGURE 2:**
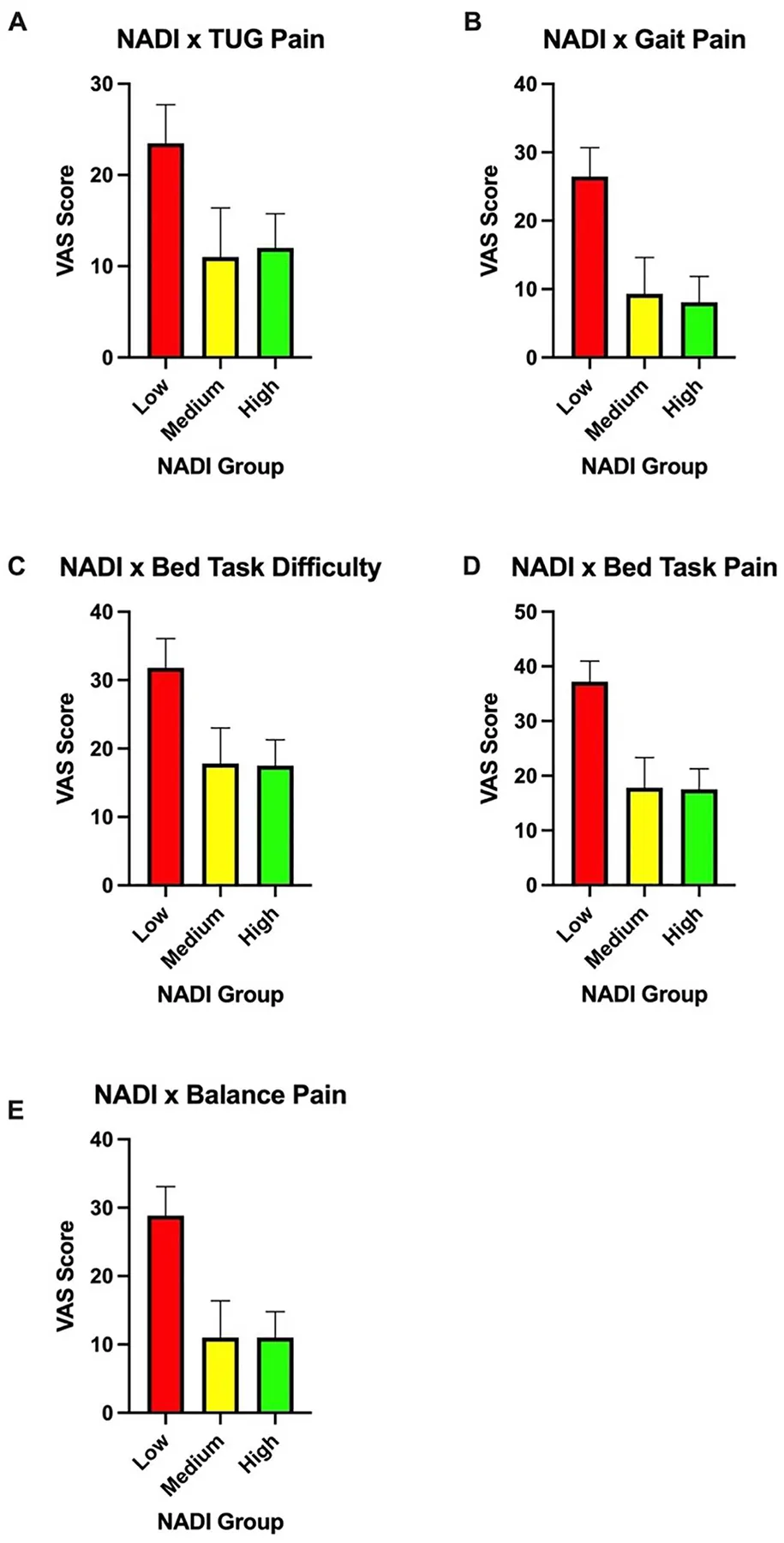
**(A)** Timed up-and-go pain scores among the three NADI groups. **(B)** Gait pain scores among the three NADI groups. **(C)** Bed-task difficulty scores among the three NADI groups. **(D)** Bed-task pain scores among the three NADI groups. **(E)** Balance pain scores among the three NADI groups.

Significant differences were observed between the pain outcomes of gait pain [*F*(2,76) = 3.793, *p* = 0.027], balance pain [*F*(2,76) = 3.851, *p* = 0.026], and box-lift pain [*F*(2,76) = 5.475, *p* = 0.006] among the three NADI groups. Trends toward differences in chair-stand pain [*F*(2,76) = 12.915, *p* = 0.072], TUG pain [*F*(2,76) = 15.458 *p* = 0.061], and bed-task pain [*F*(2,76) = 2.587, *p* = 0.082] were also noted between the high, medium, and low NADI groups. Figures 2A–E show the relationship between the three NADI groups and representative pain outcomes.

## Discussion

Chronic pain is not only increasing in prevalence in society but also amplifying the social, psychological, and economic consequences (2, 19). With an ever-increasing population, more individuals are affected by this crippling and lasting pain, highlighting the need for more detailed, accessible treatment plans without harmful side effects (20). Presently, we examined the associations between SES, dietary inflammatory profile, blood-based inflammatory biomarker levels, and pain outcomes in adults with cLBP. This study showed a significant positive correlation between the inflammatory potential of participants’ diet and the proinflammatory biomarker TNF-α, as well as movement-evoked pain outcomes, including balance pain, chair-stand pain, and bed-task pain. Similarly, SES status, as characterized by the NADI, was associated with higher levels of several biomarkers: IL-2, IL-7, IL-12, IL-12p70, and MCP4. In addition, participants with lower SES (indicated by the highest NADI tertile) demonstrated more proinflammatory dietary patterns, whereas those with higher SES exhibited the least proinflammatory diet patterns. Finally, when examining the difference in means between pain outcomes across our three SES groups, participants with low SES reported significantly greater reported movement-evoked pain during gait, balance, and box-lift tasks. Overall, low SES was associated with proinflammatory diet patterns and a heightened proinflammatory profile in adults with cLBP—a relationship that may contribute to more severe outcomes in individuals with cLBP. Our findings also suggest that interventions aimed at improving diet quality among socioeconomically disadvantaged adults may aid in cLBP management.

SES has been historically associated with many adverse health effects, such as increased morbidity, decreased life expectancy, infant mortality, and increased pain (21). Individuals with lower SES tend to have greater pain prevalence and are more sensitive to noxious stimuli (22, 23). Due to this increased sensitivity, it was hypothesized that those with higher NADI scores would report greater pain sensitivity during pain outcome tests like short-form performance battery tests. Our results mirror prior literature examining the relationship between individuals living in deprived communities and their respective pain ratings (18). Specifically, in our sample, those with lower NADI score tended to rate their pain severity higher during movement-evoked tasks reflecting a variety of everyday activities. SES is generally considered a non-modifiable risk factor at the individual level, as changes in its contributors arise from local, national, and international policies (24), which is not something an individual has direct control over. Both low and medium SES have been shown to moderately increase disease risk compared to high SES (25). Low SES is also associated with more severe pain, greater pain-related disability, and higher interference (23). Further examination of the literature has determined a relationship between chronic pain and SES, indicating that individuals facing higher levels of poverty are more likely to develop painful conditions such as cLBP (26). As SES decreases and poverty increases, access to healthcare decreases, contributing to worse outcomes (27). Individuals living in areas with lower NADI scores have also shown higher levels of alcohol abuse, another risk factor for chronic pain (10, 26). Nevertheless, diet represents an identifiable target of intervention, even among individuals in low SES groups. Food insecurity, for example, is potentially modifiable through food assistance programs such as the Supplemental Nutrition Assistance Program (SNAP), which has been shown to have a protective effect against chronic pain prevalence (28). In addition, educational interventions can help individuals with limited financial resources improve their nutrition knowledge and make more informed dietary purchases (29). Considering these findings, our results suggest that screening for poor diet quality and food insecurity in clinical settings may help healthcare providers identify adults with cLBP who would benefit from nutrition education and food assistance programs and, importantly, facilitate their connection to these resources.

A potential reason for neighborhoods having lower NADI scores is the presence of limited income or wealth, leaving residents with little to no buffer when faced with negative health outcomes (15). Lack of income can also play a role in lifestyle choices. Maintaining a healthy and active lifestyle is often costly and time-consuming—resources that may be unavailable to residents working longer hours to meet household needs. Previous literature suggests that individuals in higher socioeconomic environments tend to have healthier eating patterns, while the opposite is true of those in lower SES contexts (30). Although healthy foods are not always more expensive, they often can be, forcing individuals facing poverty to settle for less expensive options (15). Predominantly low-income sectors of US communities have significantly higher access to processed fast food (31). Due to food insecurity, limited resources, and restricted income, the diet quality among residents of highly deprived areas may play a key role in the development of chronic pain conditions such as cLBP. Processed foods are known to be less expensive, but they do not carry the same nutritional quality as whole foods. Presently, our results align with the literature in that dietary quality decreases as NADI increases, as indicated by more proinflammatory DII scores. High proinflammatory diets are typically characterized by processed carbohydrates, fats (specifically saturated and trans fatty acids), and increased alcohol consumption (32). Diet quality can contribute to inflammation through several mechanisms, the most common being elevated proinflammatory cytokine production, heightened oxidative stress, and increased sympathetic activity (33).

Inflammation is a normal response driven by the immune system, typically triggered by infection, allergy, or peripheral injury (34). During this process, several immune cells, such as macrophages and neutrophils, release proinflammatory mediators (35, 36). Of particular interest to this study, TNF-α, IL-2, IL-7, IL-12, IL-12p70, and MCP4 are inflammatory molecules associated with pain and NADI outcomes. It is hypothesized that these mediators can sensitize certain areas, such as the spinal cord, anterior cingulate cortex, and insula, contributing to the maintenance of chronic pain (37). Peripheral and central sensitization contributes to the constant presence of chronic pain by lowering the threshold of sensory and interneuron activation and signaling, potentially influencing what is interpreted by the brain as painful (36). Subsequent plastic synaptic changes can also lead to an increased neuronal response in the pain pathway after presentation with a stimulus that may usually not be perceived as painful (38). If not addressed, this sensitization of central nervous system components can subsequently lead to hyperalgesia and allodynia due to signal amplification (39, 40, 41). A Western diet high in processed foods, refined grains, sweets, and desserts is positively correlated with higher levels of proinflammatory cytokines (42). Oxidative stress occurs when there is an imbalance between the production and detoxification of reactive oxygen species (ROS), leading to their accumulation (43). ROS has been shown to activate several transcription factors that mediate genes involved in inflammation (44). Similarly, the relationship between diet and pain has been observed in many studies. An individual’s hyperalgesia and allodynia have been shown to be influenced by diet (45), meaning what a person eats can affect their experience of pain. High-fat diets, a common characteristic of the Western diet, have been shown to induce mechanical allodynia (46). Inflammation contributes to hyperalgesia and allodynia by causing hypersensitive responses to noxious and non-noxious stimuli (47). Although inflammation-induced hyperalgesia can have a protective role, prolonged effects can transform this protection into persistent pain (48). Our results are consistent with the idea that patients with chronic low back pain who have higher DII scores also exhibit increased pain sensitivity during movement-evoked tasks.

Previous literature has shown that inflammation levels are positively correlated with impoverishment (49). Our results are consistent with the idea that individuals living in poor-quality environments are at greater risk for increased inflammation levels. A lower socioeconomic lifestyle typically carries many risk factors that can contribute to inflammation, including stressful life events, lower education, less access to healthcare, unbalanced diets, less social support, and maladaptive coping mechanisms (25, 48, 49). Longer hours, physically demanding, repetitive work, and nighttime shifts are examples of lower socioeconomic job environments that can increase inflammation (50). In addition, in our study, individuals with lower SES also tended to follow more proinflammatory diet patterns, which may further influence inflammation and subsequent pain. Typical unhealthy foods that promote inflammation include trans fats, refined carbohydrates, omega-6 polyunsaturated fatty acids (PUFAs), and many others, which are prevalent in the Western diet (51). However, several dietary interventions have shown promise in decreasing inflammation. A Mediterranean diet, characterized by high consumption of fruits, vegetables, fish, olive oil, nuts, and grains, has demonstrated lower levels of inflammatory biomarkers (51, 52). The anti-inflammatory diet (AID) was designed to include ingredients like curcumin and ginseng, which have significant effects on hyperalgesia and allodynia (53). In addition, there is evidence to support the notion that dietary interventions are an effective non-pharmacological, non-surgical way to reduce inflammation and promote recovery (53). Diet plays a major role in the development of obesity, a proinflammatory state and a comorbidity in cLBP (54, 55). Greater amounts of adipose tissue and a poor-quality diet have been shown to activate the immune system, leading to increased inflammation (45). This increased inflammation has been associated with not only movement pain but also more rigid, slower movements (56). Our results align with previous literature indicating that poverty, lack of income, and low education contribute to proinflammatory effects.

The results should be interpreted in light of several study limitations. We acknowledge that using a self-reported 24-h food recall, there can be much variability; however, it should be noted that all efforts were made to train research assistants on how to probe the participant and provide visual aids. Our sample size was relatively small, and based on convenience, as only a small portion of participants from the parent study had complete data for the present analyses. Findings regarding comparisons among the three SES groups may be quite conservative, as we had sufficient power only to detect large effect sizes. Therefore, caution is warranted when interpreting and generalizing the findings. Data were collected during the COVID-19 pandemic, which raises the possibility of affecting our sample. Our pain outcomes were also self-reported, which could lead to variability despite the use of a standard rating scale. The NADI was calculated using the current address of participants, without accounting for the length of residence. Future research should explore the relationship between the length of residence and pain outcomes regarding the NADI. In addition, future research could explore the role of coping strategies and adverse childhood effects to determine their impact on pain, dietary behavior, and inflammation when using diet and NADI as variables of interest. Our research only looked at pain outcomes, inflammation levels, diet, and SES regarding chronic low back pain. Future studies should be done using other chronic pain disorders to assess whether similar results, including those related to causality, are established. In addition, research is needed to develop community-based interventions to improve healthy food access and diet quality among low SES groups with or at risk for cLBP.

The prevalence of chronic low back pain continues to increase with an ever-increasing population. Many therapies attempt to offer relief but only provide acute effects and may cause detrimental side effects. Although this study did not directly offer health benefits, it was done to not only support the goal of developing specific treatments for subgroups of the population but also identify risk factors associated with the development of this disorder to prevent its development altogether. In our study, the complex relationship between SES and dietary quality appears to affect inflammation levels and pain outcomes. These results are imperative moving forward, allowing for targeting goals toward vulnerable populations. In addition, these findings may help address specific health disparities related to diet and environmental risk factors.

## Figures and Tables

**TABLE 1:** Participant demographic characteristics stratified by NADI groups (high, medium, low).

Variable	Total participants (*N* = 78) (%)	High NADI (>66)	Medium NADI (33–66)	Low NADI (<33)	*p*-value
Sex					0.545
Female	45 (57.7)	29 (58.0)	10 (66.7)	6 (46.2)	
Male	33 (42.3)	21 (42.0)	5 (33.3)	7 (53.8)	
Race					0.001*
Black	50 (64.1)	39 (78.0)	8 (53.3)	3 (23.1)	
White	28 (35.9)	11 (22.0)	7 (46.7)	10 (76.9)	
Age (years)	44.71 ± 12.759	45.76 ± 11.969	41.07 ± 13.609	44.85 ± 14.910	0.540
BMI	30.87 ± 8.060	31.48 ± 7.552	30.53 ± 11.031	28.90 ± 6.387	0.198
NADI	69.08 ± 26.803	—	—	—	—
DII	2.10 ± 1.756	2.33 ± 1.739	1.69 ± 1.419	1.68 ± 2.111	0.006*
Education					0.014*
College graduate and above	25 (32.1)	10 (20.0)	7 (46.7)	8 (61.5)	
Partial college and below	53 (68)	40 (80.0)	8 (53.3)	5 (38.5)	
Income					<0.001*
Above poverty	45 (57.7)	20 (40.0)	12 (80.0)	13 (100.0)	
Below poverty	31 (39.7)	28 (56.0)	3 (20.0)	0 (0.00)	
Employment status					0.206
Full-time	36 (46.2)	18 (36.0)	9 (60.0)	9 (69.2)	
Part-time	11 (14.1)	8 (16.0)	1 (6.70)	2 (15.4)	
Other	31 (39.7)	24 (48.0)	5 (33.3)	2 (15.4)	

The variables demonstrate the number of participants in the said category with percent frequency in parentheses. Continuous variables are represented as means ± standard deviations. Significant *p*-values are indicated with an asterisk (*) and are bolded. The BMI variable was calculated by dividing weight by height squared.

**TABLE 2:** ANCOVA statistics for variables statistically differed by NADI groups (*α* = 95%, *p* < 0.05).

Dependent variable	High NADI, mean (CI)	Medium NADI, mean (CI)	Low NADI, mean (CI)	*F*-value (2,76)	*p*-value	Effect size	Observed power
DII	1.61 (0.57–2.66)	1.66 (0.92–2.41)	2.33 (1.83–2.82)	179.48	0.006	0.764	0.964
TNF-β	0.23 (0.16–0.30)	0.21 (0.16–0.26)	0.21 (0.18–0.25)	26.84	0.036	0.717	0.613
IP-10	369.74 (299.53–439.95)	360.01 (256.78–463.24)	385.93 (326.70–445.17)	25.60	0.038	0.578	0.991
IL-7	6.19 (4.02–8.36)	4.51 (3.01–6.01	4.21 (2.94–5.49)	73.76	0.013	0.803	0.886
Gait pain	7.37 (1.78–12.96)	9.33 (−0.48–19.14)	26.23 (17.59–34.88)	3.793	0.027	0.655	0.909
Balance pain	11.16 (5.27–17.05)	13.61 (0.91–26.31)	29.33 (20.61–38.04)	3.851	0.026	0.713	0.977
Box-lift pain	19.32 (11.41–27.22)	21.83 (8.08–35.59)	35.80 (28.56–43.04)	5.475	0.006	0.625	0.842

## Data Availability

The raw data supporting the conclusions of this article will be made available upon reasonable request.

## Abbreviations

ANOVA: analysis of variance
cLBP: chronic low back pain
DII: dietary inflammatory index
GM-CSF: granulocyte-macrophage colony-stimulating factor
IFN-*γ*: interferon gamma
IL: interleukin
IP-10: interferon gamma-inducible protein 10
MCP: monocyte chemoattractant protein
MDC: macrophage-derived chemokine
MIP: macrophage inflammatory protein
NADI: National Area Deprivation Index
NDSR: Nutrition Data System for Research
SES: socioeconomic status
SPPB: short physical performance battery
TARC: thymus and activation-regulated chemokine
TNF: tumor necrosis factor
TUG: timed up-and-go
UAB: the University of Alabama at Birmingham
VEGF-5: vascular endothelial growth factor 5

## References

[1] Cohen SP, Vase L, Hooten WM. Chronic pain: an update on burden, best practices, and new advances. Lancet. (2021) 397(10289):2082–97. doi: 10.1016/S0140-6736(21)00393-7 34062143

[2] Raja SN, Carr DB, Cohen M, Finnerup NB, Flor H, Gibson S, The revised International Association for the Study of Pain definition of pain: concepts, challenges, and compromises. Pain. (2020) 161(9):1976–82. doi: 10.1097/j.pain.0000000000001939 32694387 PMC7680716

[3] Treede RD, Rief W, Barke A, Aziz Q, Bennett MI, Benoliel R, A classification of chronic pain for ICD-11. Pain. (2015) 156(6):1003–7. doi: 10.1097/j.pain.0000000000000160 25844555 PMC4450869

[4] Allegri M, Montella S, Salici F, Valente A, Marchesini M. Compagnone C, Mechanisms of low back pain: a guide for diagnosis and therapy. F1000Res. (2016) 5: F1000. doi: 10.12688/f1000research.8105.2 PMC4926733 27408698

[5] Krismer M, van Tulder M. Strategies for prevention and management of musculoskeletal conditions. Low back pain (non-specific). Best Pract Res Clin Rheumatol. (2007) 21(1):77–91. doi: 10.1016/j.berh.2006.08.004 17350545

[6] Meier R, Emch C, Gross-Wolf C, Pfeiffer F, Meichtry A, Schmid A, Sensorimotor and body perception assessments of nonspecific chronic low back pain: a cross-sectional study. BMC Musculoskelet Disord. (2021) 22(1):391. doi: 10.1186/s12891-021-04269-7 33902545 PMC8077718

[7] Mills SEE, Nicolson KP, Smith BH. Chronic pain: a review of its epidemiology and associated factors in population-based studies. Br J Anaesth. (2019) 123(2): e273–83. doi: 10.1016/j.bja.2019.03.023 31079836 PMC6676152

[8] Turner JA, Franklin G, Fulton-Kehoe D, Egan K, Wickizer TM, Lymp JF, Prediction of chronic disability in work-related musculoskeletal disorders: a prospective, population-based study. BMC Musculoskelet Disord. (2004) 5:14. doi: 10.1186/1471-2474-5-14 15157280 PMC428578

[9] National Cancer Institute. Definition: socioeconomic status. Available online at: https://www.cancer.gov/publications/dictionaries/cancer-terms/def/socioeconomic-status (Accessed March 18, 2023).

[10] Jordan KP, Thomas E, Peat G, Wilkie R, Croft P. Social risks for disabling pain in older people: a prospective study of individual and area characteristics. Pain. (2008) 137(3):652–61. doi: 10.1016/j.pain.2008.02.030 18434022

[11] Hummer RA, Hamilton ER. “Socioeconomic status and U.S. Population health”. In: Population Health in America. Berkley, CA: University of California Press (2019). p. 97–126. doi: 10.2307/j.ctvh1dxgm.10

[12] Li W, Gong Y, Liu J, Guo Y, Tang H, Qin S, Peripheral and central pathological mechanisms of chronic low back pain: a narrative review. J Pain Res. (2021) 14:1483–94. doi: 10.2147/JPR.S306280 34079363 PMC8166276

[13] Calder PC, Ahluwalia N, Brouns F, Buetler T, Clement K, Cunningham K, Dietary factors and low-grade inflammation in relation to overweight and obesity. Br J Nutr. (2011) 106(3):S1–78. doi: 10.1017/S0007114511005460 22133051

[14] Bjørklund G, Aaseth J, Doşa MD, Pivina L, Dadar M, Pen JJ, Does diet play a role in reducing nociception related to inflammation and chronic pain? Nutrition. (2019) 66:153–65. doi: 10.1016/j.nut.2019.04.007 31301604

[15] Kern DM, Auchincloss AH, Stehr MF, Diez Roux AV, Moore LV, Kanter GP, Neighborhood prices of healthier and unhealthier foods and associations with diet quality: evidence from the multi-ethnic study of atherosclerosis. Int J Environ Res Public Health. (2017) 14(11):1394. doi: 10.3390/ijerph14111394 29144387 PMC5708033

[16] Mohammed SH, Habtewold TD, Birhanu MM, Sissay TA, Tegegne BS, Abuzerr S, Neighbourhood socioeconomic status and overweight/obesity: a systematic review and meta-analysis of epidemiological studies. BMJ Open. (2019) 9(11): e028238. doi: 10.1136/bmjopen-2018-028238 PMC6886990 31727643

[17] Strath LJ, Brooks MS, Sorge RE, Judd SE. Relationship between diet and relative risk of pain in a cross-sectional analysis of the REGARDS longitudinal study. Pain Manag. (2022) 12(2):168–79. doi: 10.2217/pmt-2021-0048 34431328 PMC8772533

[18] Rumble DD, O’Neal K, Overstreet DS, Penn TM, Jackson P, Aroke EN, Sleep and neighborhood socioeconomic status: a micro longitudinal study of chronic low-back pain and pain-free individuals. J Behav Med. (2021) 44(6):811–21. doi: 10.1007/s10865-021-00234-w 34106368 PMC8595521

[19] Yong RJ, Mullins PM, Bhattacharyya N. Prevalence of chronic pain among adults in the United States. Pain. (2022) 163(2):e328–32. doi: 10.1097/j.pain.0000000000002291 33990113

[20] Mauck MC, Aylward AF, Barton CE, Birckhead B, Carey T, Dalton DM, Evidence-based interventions to treat chronic low back pain: treatment selection for a personalized medicine approach. Pain Rep. (2022) 7(5):e1019. doi: 10.1097/PR9.0000000000001019 36203645 PMC9529058

[21] Poleshuck EL, Green CR. Socioeconomic disadvantage and pain. Pain. (2008) 136(3):235–8. doi: 10.1016/j.pain.2008.04.003 18440703 PMC2488390

[22] Mechlin B Lower socioeconomic status is associated with rating experimental pain as more intense. J Pain. (2012) 13(4):S52. doi: 10.1016/j.jpain.2012.01.218

[23] Janevic MR, McLaughlin SJ, Heapy AA, Thacker C, Piette JD. Racial and socioeconomic disparities in disabling chronic pain: findings from the health and retirement study. J Pain. (2017) 18(12):1459–67. doi: 10.1016/j.jpain.2017.07.005 28760648 PMC5682226

[24] Prego-Domínguez J, Khazaeipour Z, Mallah N, Takkouche B. Socioeconomic status and occurrence of chronic pain: a meta-analysis. Rheumatology. (2021) 60(3):1091–105. doi: 10.1093/rheumatology/keaa758 33276382

[25] Stringhini S, Carmeli C, Jokela M, Avendaño M, Muennig P, Guida F, Socioeconomic status and the 25×25 risk factors as determinants of premature mortality: a multicohort study and meta-analysis of 1·7 million men and women. Lancet. (2017) 389(10075):1229–37. doi: 10.1016/S0140-6736(16)32380-7 28159391 PMC5368415

[26] Raza A, Claeson M, Magnusson Hanson L, Westerlund H, Virtanen M, Halonen JI. Home and workplace neighborhood socioeconomic status and behavior-related health: a within-individual analysis. Ann Behav Med. (2021) 55(8):779–90. doi: 10.1093/abm/kaaa116 33580661 PMC8311784

[27] McMaughan DJ, Oloruntoba O, Smith ML. Socioeconomic status and access to healthcare: interrelated drivers for healthy aging. Front Public Health. (2020) 8:231. doi: 10.3389/fpubh.2020.00231 32626678 PMC7314918

[28] Tamargo JA, Strath LJ, Karanth SD, Spector AL, Sibille KT, Anton S, Food insecurity is associated with chronic pain and high-impact chronic pain in the USA. Public Health Nutr. (2023) 27(1):e7. doi: 10.1017/S1368980023002732 38087858 PMC10830368

[29] Bessems KMHH, Linssen E, Lomme M, van Assema P. The effectiveness of the good affordable food intervention for adults with low socioeconomic status and small incomes. Int J Environ Res Public Health. (2020) 17(7):2535. doi: 10.3390/ijerph17072535 32272792 PMC7178221

[30] Alkerwi A, Vernier C, Sauvageot N, Crichton GE, Elias MF. Demographic and socioeconomic disparity in nutrition: application of a novel correlated component regression approach. BMJ Open. (2015) 5(5):e006814. doi: 10.1136/bmjopen-2014-006814 PMC4431064 25967988

[31] Block JP, Scribner RA, DeSalvo KB. Fast food, race/ethnicity, and income: a geographic analysis. Am J Prev Med. (2004) 27(3):211–7. doi: 10.1016/j.amepre.2004.06.007 15450633

[32] Strath LJ, Sims AM, Overstreet DS, Penn TM, Bakshi RJ, Stansel BK, Dietary inflammatory index (DII) is associated with movement-evoked pain severity in adults with chronic low back pain: sociodemographic differences. J Pain. (2022) 23(8):1437–47. doi: 10.1016/j.jpain.2022.03.237 35417792 PMC9356984

[33] Kiecolt-Glaser JK. Stress, food, and inflammation: psychoneuroimmunology and nutrition at the cutting edge. Psychosom Med. (2010) 72(4):365–9. doi: 10.1097/PSY.0b013e3181dbf489 20410248 PMC2868080

[34] Medzhitov R. Origin and physiological roles of inflammation. Nature. (2008) 454(7203):428–35. doi: 10.1038/nature07201 18650913

[35] Ji R-R, Chamessian A, Zhang Y-Q. Pain regulation by non-neuronal cells and inflammation. Science. (2016) 354(6312):572–7. doi: 10.1126/science.aaf8924 27811267 PMC5488328

[36] Ji R-R, Nackley A, Huh Y, Terrando N, Maixner W. Neuroinflammation and central sensitization in chronic and widespread pain. Anesthesiology. (2018) 129(2):343–66. doi: 10.1097/ALN.0000000000002130 29462012 PMC6051899

[37] Strath LJ, Totsch SK, Quinn TL, Menard M, George AP, Lukens SL, The effect of the Standard American Diet on Iba-1 immunoreactivity in the spinal cord before and after peripheral inflammatory injury in rats. PharmaNutrition. (2021) 18:100278. doi: 10.1016/j.phanu.2021.100278

[38] Garland EL. Pain processing in the human nervous system: a selective review of nociceptive and biobehavioral pathways. Prim Care. (2012) 39(3):561–71. doi: 10.1016/j.pop.2012.06.013 22958566 PMC3438523

[39] Koch A, Zacharowski K, Boehm O, Stevens M, Lipfert P, Von Giesen HJ, Nitric oxide and pro-inflammatory cytokines correlate with pain intensity in chronic pain patients. Inflamm Res. (2007) 56(1):32–7. doi: 10.1007/s00011-007-6088-4 17334668

[40] Crofford LJ. Chronic pain: where the body meets the brain. Trans Am Clin Climatol Assoc. (2015) 126:167–83. 26330672 PMC4530716

[41] Marchand F, Perretti M, McMahon SB. Role of the immune system in chronic pain. Nat Rev Neurosci. (2005) 6(7):521–32. doi: 10.1038/nrn1700 15995723

[42] Lopez-Garcia E, Schulze MB, Fung TT, Meigs JB, Rifai N, Manson JE, Major dietary patterns are related to plasma concentrations of markers of inflammation and endothelial dysfunction. Am J Clin Nutr. (2004) 80(4):1029–35. doi: 10.1093/ajcn/80.4.1029 15447916

[43] Pizzino G, Irrera N, Cucinotta M, Pallio G, Mannino F, Arcoraci V, Oxidative stress: harms and benefits for human health. Oxid Med Cell Longev. (2017) 2017:8416763. doi: 10.1155/2017/8416763 28819546 PMC5551541

[44] Hussain T, Tan B, Yin Y, Blachier F, Tossou MCB, Rahu N. Oxidative stress and inflammation: what polyphenols can do for us? Oxid Med Cell Longev. (2016) 2016:7432797. doi: 10.1155/2016/7432797 27738491 PMC5055983

[45] Totsch SK, Waite ME, Sorge RE. Dietary influence on pain via the immune system. Prog Mol Biol Transl Sci. (2015) 131:435–69. doi: 10.1016/bs.pmbts.2014.11.013 25744682

[46] Tierney JA, Uong CD, Lenert ME, Williams M, Burton MD. High-fat diet causes mechanical allodynia in the absence of injury or diabetic pathology. Sci Rep. (2022) 12(1):14840. doi: 10.1038/s41598-022-18281-x 36050326 PMC9437006

[47] He Y, Kim PY. “Allodynia”. In: StatPearls. St. Petersburg, FL: StatPearls Publishing (2018).

[48] Lolignier S, Eijkelkamp N, Wood JN. Mechanical allodynia. Pflugers Arch. (2015) 467(1):133–9. doi: 10.1007/s00424-014-1532-0 24846747 PMC4281368

[49] Muscatell KA, Brosso SN, Humphreys KL. Socioeconomic status and inflammation: a meta-analysis. Mol Psychiatry. (2020) 25(9):2189–99. doi: 10.1038/s41380-018-0259-2 31628416 PMC6814496

[50] Kokosi T, Flouri E, Midouhas E. Do upsetting life events explain the relationship between low socioeconomic status and systemic inflammation in childhood? Results from a longitudinal study. Brain Behav Immun. (2020) 84:90–6. doi: 10.1016/j.bbi.2019.11.013 31756384

[51] Schwingshackl L, Morze J, Hoffmann G. Mediterranean diet and health status: active ingredients and pharmacological mechanisms. Br J Pharmacol. (2020) 177(6):1241–57. doi: 10.1111/bph.14778 31243760 PMC7056467

[52] Mena MP, Sacanella E, Vazquez-Agell M, Morales M, Fito M, Escoda R, Inhibition of circulating immune cell activation: a molecular antiinflammatory effect of the Mediterranean diet. Am J Clin Nutr. (2009) 89(1):248–56. doi: 10.3945/ajcn.2008.26094 19056596

[53] Totsch SK, Meir RY, Quinn TL, Lopez SA, Gower BA, Sorge RE. Effects of a standard American diet and an anti-inflammatory diet in male and female mice. Eur J Pain. (2018) 22(7):1203–13. doi: 10.1002/ejp.1207 29436058

[54] McCarthy LH, Bigal ME, Katz M, Derby C, Lipton RB. Chronic pain and obesity in elderly people: results from the Einstein aging study. J Am Geriatr Soc. (2009) 57(1):115–9. doi: 10.1111/j.1532-5415.2008.02089.x 19054178 PMC2763486

[55] Dinsa GD, Goryakin Y, Fumagalli E, Suhrcke M. Obesity and socioeconomic status in developing countries: a systematic review. Obes Rev. (2012) 13(11):1067–79. doi: 10.1111/j.1467-789X.2012.01017.x 22764734 PMC3798095

[56] Lasselin J, Sundelin T, Wayne PM, Olsson MJ, Göranson SP, Axelsson J, Biological motion during inflammation in humans. Brain Behav Immun. (2020) 84:147–53. doi: 10.1016/j.bbi.2019.11.019 31785395 PMC7010549

[57] Hébert JR, Shivappa N, Wirth MD, Hussey JR, Hurley TG. Perspective: the dietary inflammatory index (DII)-lessons learned, improvements made, and future directions. Adv Nutr. (2019) 10(2):185–95. doi: 10.1093/advances/nmy071 30615051 PMC6416047

